# The putative vacuolar processing enzyme gene *TaVPE3cB* is a candidate gene for wheat stem pith-thickness

**DOI:** 10.1007/s00122-023-04372-4

**Published:** 2023-05-26

**Authors:** Qier Liu, Yun Zhao, Shanjida Rahman, Maoyun She, Jingjuan Zhang, Rongchang Yang, Shahidul Islam, Graham O’Hara, Rajeev K. Varshney, Hang Liu, Hongxiang Ma, Wujun Ma

**Affiliations:** 1 Centre for Crop and Food Innovation, Food Futures Institute and College of Science, Health, Engineering and Education, Murdoch University, Perth, WA 6150 Australia; 2 Institute of Cereal and Oil Crops, Hebei Academy of Agriculture and Forestry Sciences, Shijiazhuang, 050035 People’s Republic of China; 3 College of Agronomy, Qingdao Agriculture University, Qingdao, 266109 People’s Republic of China; 4 Provincial Key Laboratory of Agrobiology, and Institute of Food Crops, Jiangsu Academy of Agricultural Sciences, Nanjing, 210014 People’s Republic of China

## Abstract

**Key message:**

The vacuolar processing enzyme gene *TaVPE3cB* is identified *as a* candidate gene for a QTL of wheat pith-thickness on chromosome 3B by BSR-seq and differential expression analyses.

**Abstract:**

The high pith-thickness (PT) of the wheat stem could greatly enhance stem mechanical strength, especially the basal internodes which support the heavier upper part, such as upper stems, leaves and spikes. A QTL for PT in wheat was previously discovered on 3BL in a double haploid population of ‘Westonia’ × ‘Kauz’. Here, a bulked segregant RNA-seq analysis was applied to identify candidate genes and develop associated SNP markers for PT. In this study, we aimed at screening differentially expressed genes (DEGs) and SNPs in the 3BL QTL interval. Sixteen DEGs were obtained based on BSR-seq and differential expression analyses. Twenty-four high-probability SNPs in eight genes were identified by comparing the allelic polymorphism in mRNA sequences between the high PT and low PT samples. Among them, six genes were confirmed to be associated with PT by qRT-PCR and sequencing. A putative vacuolar processing enzyme gene *TaVPE3cB* was screened out as a potential PT candidate gene in Australian wheat ‘Westonia’. A robust SNP marker associated with *TaVPE3cB* was developed, which can assist in the introgression of *TaVPE3cB.b* in wheat breeding programs. In addition, we also discussed the function of other DEGs which may be related to pith development and programmed cell death (PCD). A five-level hierarchical regulation mechanism of stem pith PCD in wheat was proposed.

**Supplementary Information:**

The online version contains supplementary material available at 10.1007/s00122-023-04372-4.

## Introduction

Wheat is the most widely grown crop in the world, accounting for 220 million hectares with annual global production of ∼772 million tonnes (FAOSTAT 2022). By 2050, global demand for wheat is predicted to grow sharply as the world’s population is expected to exceed 9 billion (Keating et al. 2014). The Green Revolution led to tremendous increases in wheat yield by providing excellent growing conditions and improving crop varieties. With advances in molecular genetics technology, some yield-related genes that control plant height and tiller number have been cloned, such as wheat reduced-height genes (*Rht*) (Appleford et al. 2007) and semi-dwarf gene (*Sd1*) (Monna et al. 2002). The semi-dwarf cultivars are inherently more stable mechanically, reducing the leverage on the stem base and anchorage system in wheat, thereby increasing the lodging resistance under nitrogen application and achieving maximum yield potential (Hedden 2003). However, severe dwarfism causes inadequate biomass accumulation, eventually, lower yield potential (Hirano et al. 2017). Therefore, breeding wheat varieties with strong stem phenotypes is a breeding strategy for enhancing lodging resistance and yield (Reynolds et al. 2010).

The wheat stem plays an important role in providing mechanical support for leaves and spikes (Kirby 2002), transporting water and mineral nutrients, storing water-soluble carbohydrates (WSC) and starch (Scofield et al. 2009), and remobilizing nutrients during grain filling (Blum 1998). Stem lodging is mainly occurring at the 2nd internode (Peng et al. 2014), and the density of the basal 2nd internode has been proven to correlate with stem mechanical strength (Li et al. 2022). The central part of the young stem is occupied by pith tissues, which are composed of undifferentiated parenchyma cells. Parenchymatous pith cells store a large amount of water and WSCs, such as sucrose, glucose, fructose and fructan (Ruuska et al. 2006). In mature wheat stems, the majority of pith cells die and collapse, which leads to the formation of a central cavity and hollow stem. The death of the pith cells has been regarded as programmed cell death (PCD), but the molecular mechanism of pith death remains unexplained (Fujimoto et al. 2018).

Stem pith thickness is an important agronomic trait of durum and bread wheat that provides resistance to the wheat pest (Hayat et al. 1995), lodging (Kong et al. 2013) and drought (Saint Pierre et al. 2010). Adopting forward genetic strategies, many stem- strength-related QTLs have been identified on 1A and 2D for culm wall thickness (Hai et al. 2005; Liu et al. 2017; Pan et al. 2017); 3B and 2D for culm diameter (Hai et al. 2005; Song et al. 2021); 1A, 3A and 4B for stem internode length (Berry et al. 2008; Piñera‐Chavez et al., 2021); 1B, 2D, 3A, 3B, 4B and 4D for stem internode wall width (Berry et al. 2008; Piñera‐Chavez et al., 2021; Verma et al. 2005). The major genetic factor related to stem solidness has been mapped on chromosome 3B in durum (*SSt1*) and bread wheat (*Qss.msub-3BL*), conferring solid stems with thick sclerenchyma tissues and a strong culm phenotype (Cook et al. 2004; Nilsen et al. 2017). Recently, a putative Dof transcription factor, *TdDof*, was cloned as the *SSt1* causal gene (Nilsen et al. 2020).

BSR-seq approach that combines bulked segreant analysis (BSA) with RNA sequencing provides an efficient method to rapidly identify candidate genes of QTLs. It uses RNA sequencing data to call SNPs and filter out SNPs linked to the candidate genomic region through BSA, thus the hot spot region of genetic variation associated with the phenotype could be identified (Liu et al. 2012). In addition, RNA-seq reveals DEGs between two bulked sample pools in the mapping interval and provides the necessary information for gene screening. Recently, several genes have been identified through BSR-seq in different plant species, including the genes related to powdery mildew resistance (Xie et al. 2020; Zhan et al. 2021), pest resistance (Hao et al. 2019), male sterile (Tan et al. 2019) and waterlogging-tolerance (Du et al. 2017).

The objectives of this study were to: (i) identify genome-wide mRNA variants related to PT through BSR-seq; (ii) determine the physical location of *Qpt-3B* through BSR-seq; (iv) identify the candidate gene for *Qpt-3B*; (v) develop SNP marker linked to *Qpt-3B* for marker-assisted selection.

## Materials and methods

**Fig. 1:**
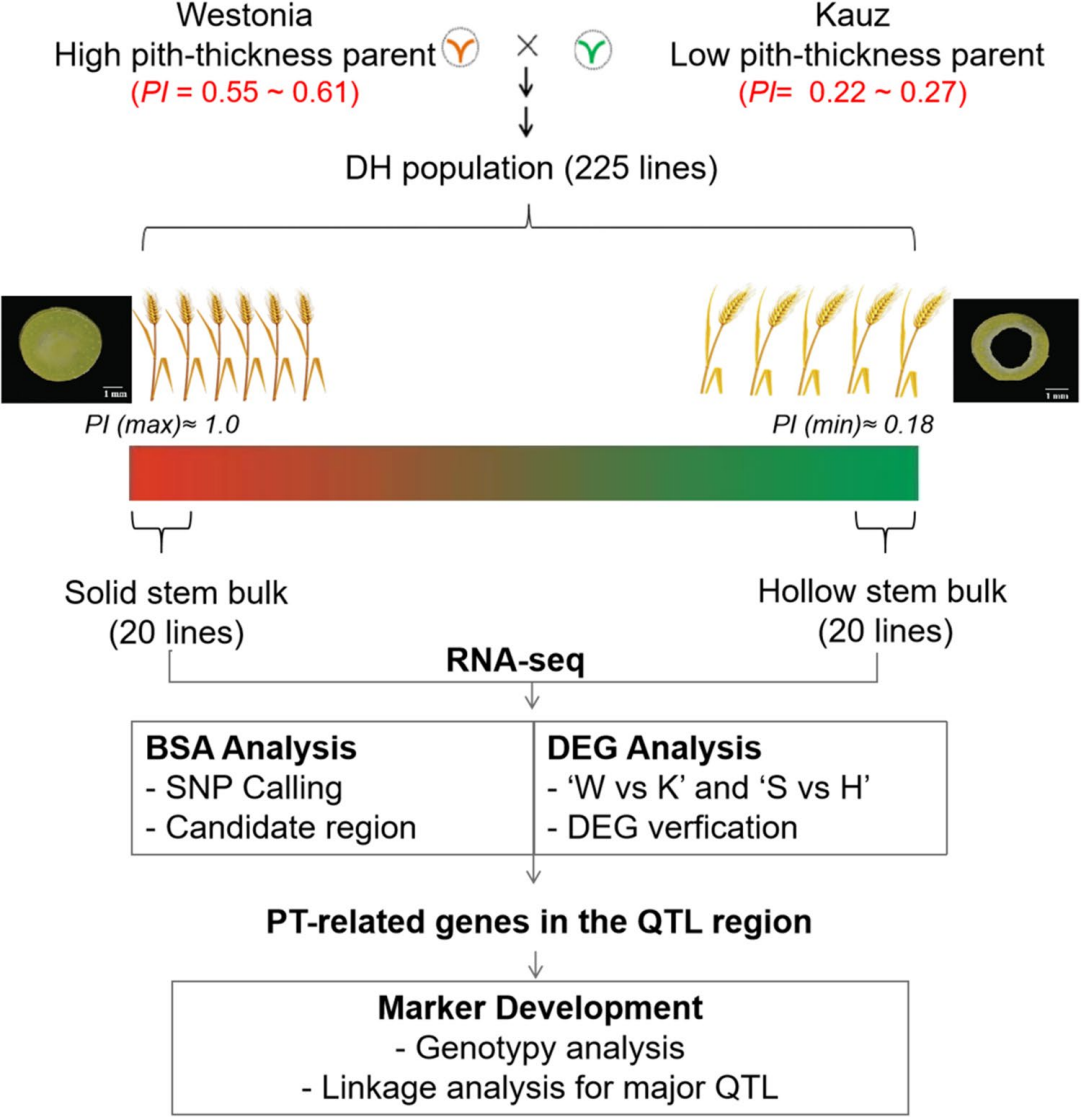
Schematic flowchart of the experimental procedure. W: ‘Westonia’; K: ‘Kauz’; S: Solid bulks; H: Hollow bulks

The overall experimental procesure is outlined in Fig. 1.

### Plant materials and growth conditions

A doubled haploid (DH) population ‘Westonia’ (high PT) x ‘Kauz’ (low PT) with 225 lines were used for the PT candidate gene identification (Butler et al. 2005; Rajaram et al. 2002; Zhang et al. 2013). A set of Australian historical wheat cultivar collections (171 varieties) spanning approximately 125 years (1890–2015) was selected for marker validation (Table S1). The genetic resource information can be found in the CIMMYT-Wheat Germplasm Bank (https://wgb.cimmyt.org/gringlobal/search).

The DH population and historical cultivars were grown repeatedly in a glasshouse at Murdoch University, Western Australia, Australia, from 2018 to 2020. Pots were placed following a complete randomized block design (RCBD) and three seeds from each cultivar were planted in a 4 L free-draining pot filled with soil mix. Plants were grown under controlled temperature with 25/15 °C (day/night) and sunlight conditions and equipped with an automatic watering system.

### Evaluation of stem pith thickness

Pith-thickness data of each line was obtained at the fully matured stage (Pluta et al. 2021) by evaluating the average rating of the stem: the main stem was cross-sectioned in the center of the second basal internode, and the stem diameter and pith thickness were measured by the Vernier caliper with three biological replicates. The pith filling of internode was rated using a five-grade system according to the methodology developed by PAUW and Read (1982). Pith-thickness index was calculated with the formula: PI = (2 × pith thickness)/stem diameter (Wallace et al. 1973).

### RNA isolation, library construction and sequencing

The selected DH lines (listed in Table S2) and two parental lines were used for RNA-seq analysis. For each line, approximately 0.5 cm of the middle of the second basal internode of main stem was sampled at the early internode elongation stage (Z32) (Zadoks et al. 1974). RNA was extracted using the TRIzol reagent (Invitrogen Canada, catalogue No. 15596026) and subsequently treated with Qiagen DNase set (Catalog No. 79254) to remove genomic DNA. For bulked sample pooling, equal amounts of RNA from each of the 20 selected DH lines were mixed to construct solid bulk (Sbulk, the high PT lines) or hollow bulk (Hbulk, the low PT lines). Two parents and two bulked samples with three replicates each were prepared and submitted to Singapore Novogene company for sequencing.

### SNP calling and ΔSNP-index analysis

The data were ordered and assembled using SAMtools v-1.14 (http://www.htslib.org/download/). The sequencing data of three biological replicates per sample were analyzed together. The initial SNP calling was performed using Genome Analysis Toolkit (GATK, v4.2.3.0) package (McKenna et al. 2010), and SnpEff was used for SNP annotation (Cingolani et al. 2012). The high-quality SNPs were filtered according to Liu et al. (2012) with the following criteria: sequencing depth for each SNP ≥ 5; Quality of variation detection ≥ 50; the minimum quality score of 20, and only homozygous SNPs between parental lines were used for SNP-index analysis. After filtration, for each genomic position, the SNP-index of two bulks were estimated using a MutMap method, with SNPs in ‘Kauz’ as a reference. SNP-index calculates the proportion of short reads that cover a particular site sharing an SNP (Abe et al. 2012). Then, the ΔSNP index was calculated by subtracting the SNP index of the Hbulk from the Sbulk (Takagi et al. 2013). The average value of ΔSNP index in the corresponding window was plotted by calculating in a 5 Mb window size and 50 kb window step size. PT-associated loci were identified when the fitted values of ΔSNP index were higher than the 99% confidence threshold. A Circos graph (Krzywinski et al. 2009) including chromosomes, genes and SNP density was generated by CIRCOS software (http://circos.ca/).

### Differentially expressed gene analysis

The high-quality reads were mapped against the latest Chinese spring genome (IWGSC RefSeq v2.1) using the HISAT2 software (Kim et al. 2019), and the expression level was calculated with fragments per kilobase of transcript per million fragments mapped (FPKM) (Trapnell et al. 2010). The fold change was calculated based on the normalized expression values between the high PT sample and the low PT sample. Genes with more than two-fold differential expression (|fold change| ≧ 2) and false discovery rate (FDR) < 0.001 for the groups of ‘Westonia vs Kauz’ and ‘Sbulk vs Hbulk’ were classified as significant DEGs. Only the DEGs coexisting between parent comparison and bulks comparison group were considered as pith-thickness-related genes. Then, those coexisting DEGs were classified into two types, Hcluster and Scluster. Hcluster contains genes which were highly expressed in low PT samples, while Scluster consists of the genes which were highly expressed in high PT samples.

### Go and KEGG pathway analyses

Gene ontology (GO) and KEGG pathway enrichment analysis of the DEGs was performed according to the method described by Hao et al. (2019). The GO enrichment analysis was performed using an R package based on hypergeometric distribution test to find the significantly enriched terms in DEGs. For KEGG pathway enrichment analysis, the metabolic pathway annotation was performed using KOBAS software against the KEGG database (http://www.genome.jp/kegg/). GO terms and KEGG pathway with FDR corrected *p* value ≤ 0.05 was regarded as significantly enriched.

### NBT staining

The production of ROS in stems was detected by nitrotetrazolium blue chloride (NBT) staining as described by Wohlgemuth et al. (2002) with minor modifications. Stems at three different stages (Z30, Z32 and Z65) were harvested, and immersed in 50 mm PBS buffer (pH 7.8) containing 0.1 mg ml^−1^ NBT and 10 mm sodium azide. Samples were vacuum-infiltrated for 2 min, and subsequently incubated at 25 °C for 2 h in the darkness and then the stained samples were immersed in 80% (v/v) ethanol for 1 h to remove the chlorophyll. ROS production was visualised as a dark blue formazan deposit in stem tissues.

### qRT-PCR validation

qRT-PCR was performed to evaluate the reliability of the sequencing results and reveal expression profiles of DEGs. For the evaluation of sequencing results, the same RNA samples were used for qRT-PCR as for RNA-seq. In addition, the stems on three different Zadoks stages (Z30, Z32, Z65) and the leaves at the stage of Z32 were collected from parents. For *TaVPEcB* gene expression analysis of Chinese spring and the selected historical lines, the stems were collected on the stage of Z32 only. The first strand cDNA was synthesised using the SensiFAST cDNA Synthesis Kit (Bioline, UK) and the qRT-PCR amplification was performed using SensiFAST SYBR No-ROX Kit (Bioline, UK). *Taactin* was used as an internal control gene for the normalization of gene expression studies (Wang et al. 2013). The processing for the 3-step cycling qRT-PCR was as follows: 95 °C for 2 min, followed by 40 cycles of 95 °C for 5 s, 60 °C for 15 s, 72 °C for 15 s. Reaction specificities were assessed by melting curve analysis. Gene relative expression level was calculated using 2^−ΔΔCt^ method with three technical repeats (Livak and Schmittgen 2001). A one-way ANOVA followed by a Tukey’s test was performed to identify significant differences.

### Cloning, sequencing and phylogenetic analysis

The synthesized cDNA, gDNA and genome-specific primers (Table S3) were used for the amplification of full-length CDS and promoter region in both parents. The PCR reaction was conducted by using Q5 High-fidelity DNA Polymerase (NEB) according to product instructions. The target fragments were separated and purified using a Gel Extraction Kit (Promega). Then, the purified products were amplified using BigDye Terminator V3.1 Cycle Sequencing Kits (Applied Biosystems) and sequenced by Applied Biosystems 3730 DNA Analyzers.

Protein sequences of *VPE* family of *Arabidopsis*, *brachypodium*, rice and wheat were gathered from the published database, and corresponding accession numbers of used sequences are provided in Table S4. The alignment of protein sequences was performed by the ClustalX program. The phylogenetic tree was constructed by the neighbour-joining method with 1000 bootstraps in the MEGA11 software (Tamura et al. 2007). The sequence alignment and the GC content were analyzed using DNAMAN software, and their cis-acting elements were predicted by PLACE and PlantCARE.

### Maker development and linkage map construction

Co-dominant markers were designed based on the SNPs within the candidate DEGs and genotyped the 225 DH lines for being mapped onto an existing linkage map. PCR reactions were carried out in 10 μL reaction mixture consisting of GoTaq® Green Master Mix 2X (Promega), primer sets (0.5 µM), and genomic DNA (50 ng). The procedure of PCR was as follows: 95 °C for 2 min; 30 cycles of 95 °C for 10 s, and 56 °C for 15 s, 72 °C for 1 min; 72 °C for 7 min. Based on our previous mapping results of major pith thickness QTL on 3BL (Zhao 2019), the developed makers as well as the previous makers were used for a new linkage map construction with the software IciMapping software V4.1 (Meng et al. 2015). The graphical presentation of linkage maps and QTLs were conducted by MapChart V2.3.2.

## Results

### Pith-thickness evaluation of wheat lines

**Fig. 2:**
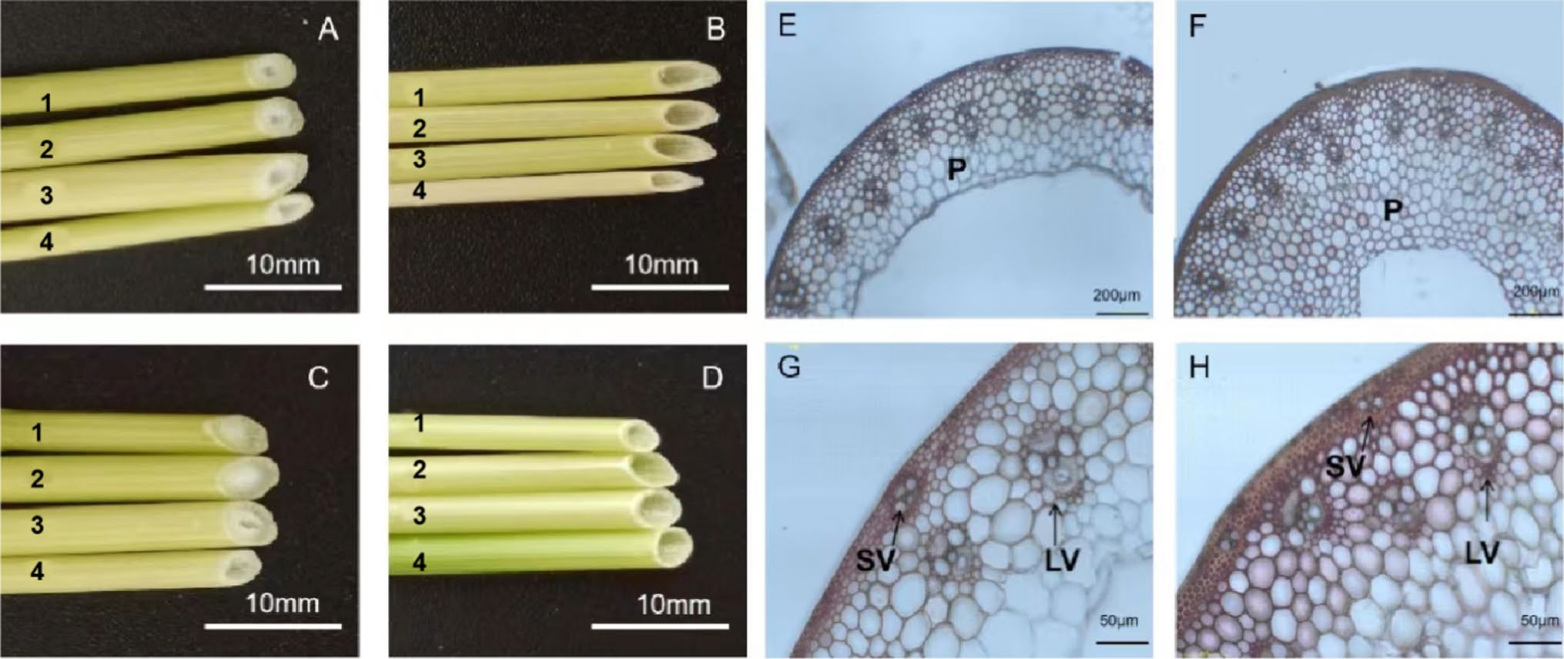
Morphological characteristics of different wheat genotypes. **A** and **C**: High pith-thickness stems ‘Westonia’ and DH line 209. **B** and **D**: hollow stem internodes of Kauz and DH line 156. Numbers 1, 2, 3 and 4 are the second, third, fourth and fifth stem internodes (from the bottom to top), respectively. **E** and **G**: Wiesner staining of the 2nd basal internode stem sections of ‘Kauz’; **F** and **H**: Wiesner staining of the 2nd basal internode stem sections of ‘Westonia’. P: pith; SV: small vascular bundle; LV: large vascular bundle

The wheat stem pith-thickness index scores varied from 0.19 to 1 (*p* < 0.05) in the DH population. Its distribution pattern was similar to a bimodal distribution (Fig. S1), consistent with single gene inheritance for stem pith thickness. Wheat parent ‘Westonia’ was classified as high PT stem (PI = 0.55~0.61) and ‘Kauz’ was classified as low PT stem (PI = 0.22~0.27) across three test years. PT index for solid bulks (Sbulks) ranged from 1.00 to 0.75, indicating that these bulked samples belong to completely solid or high PT grade, while PT index for hollow bulks (Hbulks) ranged from 0.19 to 0.25, belonging to hollow or low PT grade (Fig. 2 and Table S2).

### Differentially expressed gene identification and GO/KEGG pathway analysis

**Fig. 3:**
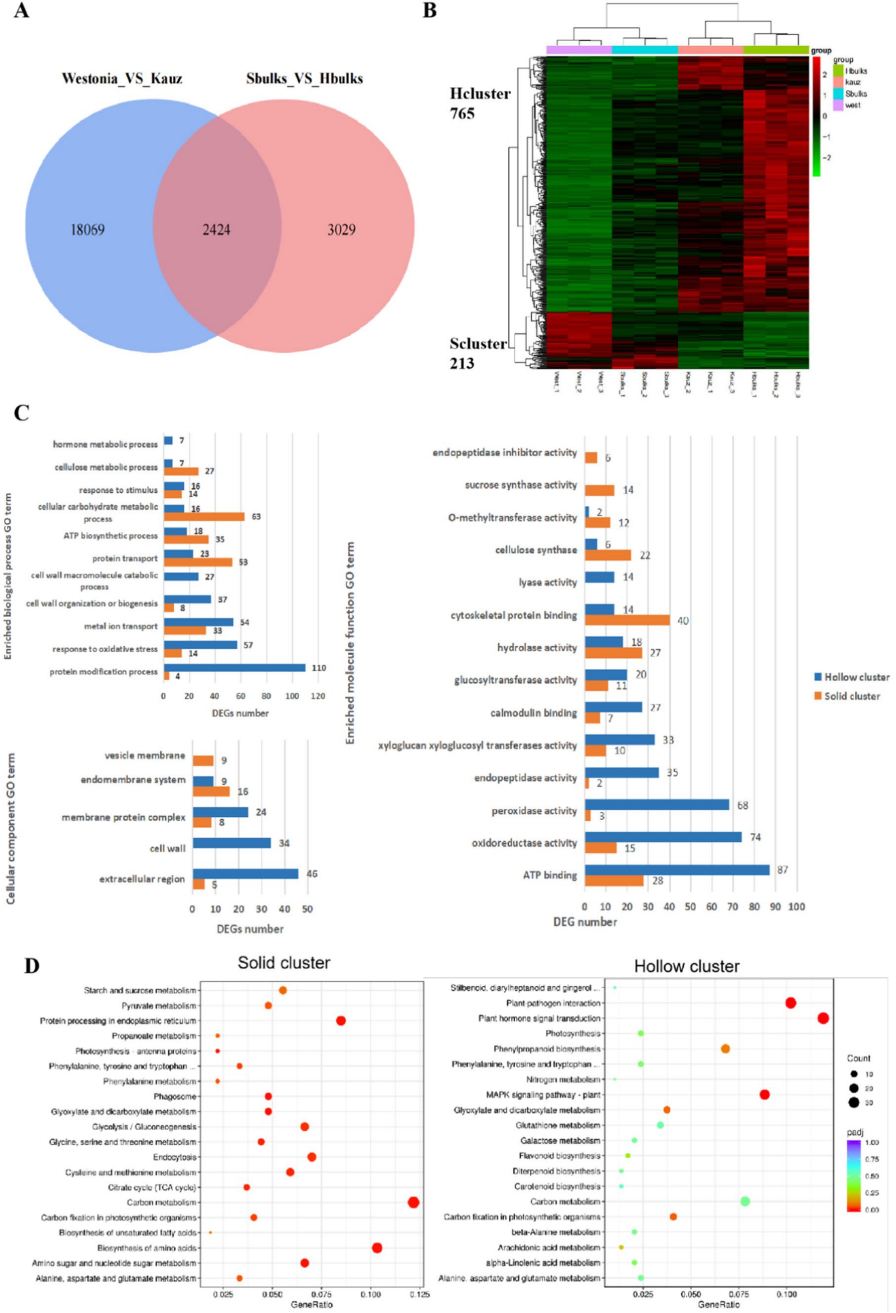
Transcriptional changes in solid stem samples (Westonia and Sbulks) and hollow stem samples (Kauz and Hbulks). **A** Venn diagram showing a total of 2424 coexisting in the comparison of ‘Westonia vs Kauz’ and ‘Sbulks vs Hbulks’; **B** Heat map showing the DEGs with the same expression profiles. Gene expression was normalized and transformed by log10 (FPKM + 1) values. Red and Green lines represent genes showing high and low expression levels, respectively. Hcluster: hollow cluster; Scluster: solid cluster; **C** GO term enrichment analysis of DEGs in two clusters; **D** Enriched KEGG pathway scatterplots for DEGs in two cluster (colour figure online)

The transcript profiles of solid and hollow stem pools were built by comparing their gene expression levels based on FPKM (fragments per kilobase of transcript per million fragments mapped). A total of 20,493 DEGs were revealed among ‘Westonia’ versus ‘Kauz’. Among them, 8209 genes were highly expressed in Westonia with 878 genes up-regulated by over 50 folds. The number of highly expressed genes in Kauz was 12,284, among which 709 genes were down-regulated by over 50 folds. However, only 5453 DEGs were identified between Sbulk versus Hbulk. Among them, 1613 genes were up-regulated and 3840 were down-regulated, with 93 and 256 genes up-regulated and down-regulated by 20–50 folds, respectively. In addition, in this comparison group, no DEGs with more than 50-fold difference has been found. The number of DEGs in the two comparison groups differed significantly, but 2424 common DEGs were identified (Fig. 3A). Among the coexisting DEGs, 765 genes were classified as hollow cluster (Hcluster) genes which were highly expressed in all low PT samples (Fig. 3B) and 213 genes were classified as solid cluster (Scluster) genes which were highly expressed in all high PT samples.

Results from GO analysis on the DEGs showed that in the biological process, Scluster DEGs were mainly enriched in cellular carbohydrate metabolic process, protein transport and ATP biosynthetic process (Fig. 3C). However, Hcluster DEGs were mainly involved in the protein modification process, response to oxidative stress, metal ion transport and cell wall organization or biogenesis. In addition, the top enriched molecule functions in Scluster were associated with cytoskeletal protein binding, ATP binding, hydrolase activity and transferase activity (such as glucosyltransferase and *O*-methyltransferase activity; while the most enriched GO terms in Hcluster were ATP binding, oxidoreductase activity, peroxidase activity and endopeptidase activity. The most enriched cellular components in Scluster belonged to the membrane protein complex, endomembrane system and vesicle membrane; while in Hcluster, the extracellular region and cell wall were significantly enriched.

KEGG pathway analysis showed that the significantly enriched metabolic pathways in the Scluster including carbon metabolism, biosynthesis of amino acids and protein processing (Fig. 3D). While in the Hcluster, four significantly enriched pathways were found, including the plant-pathogen interaction pathway, plant hormone signal transduction, phenylpropanoid biosynthesis and MAPK signalling pathway. Among them, phenylpropanoid biosynthesis is an important metabolic pathway to scavenge the over-accumulated reactive oxygen species (ROS) which may cause oxidative damage to proteins, lipids, and DNA, ultimately resulting in PCD (Sharma et al. 2012).

### Histochemical detection of superoxide anion accumulation during stem development

**Fig. 4:**
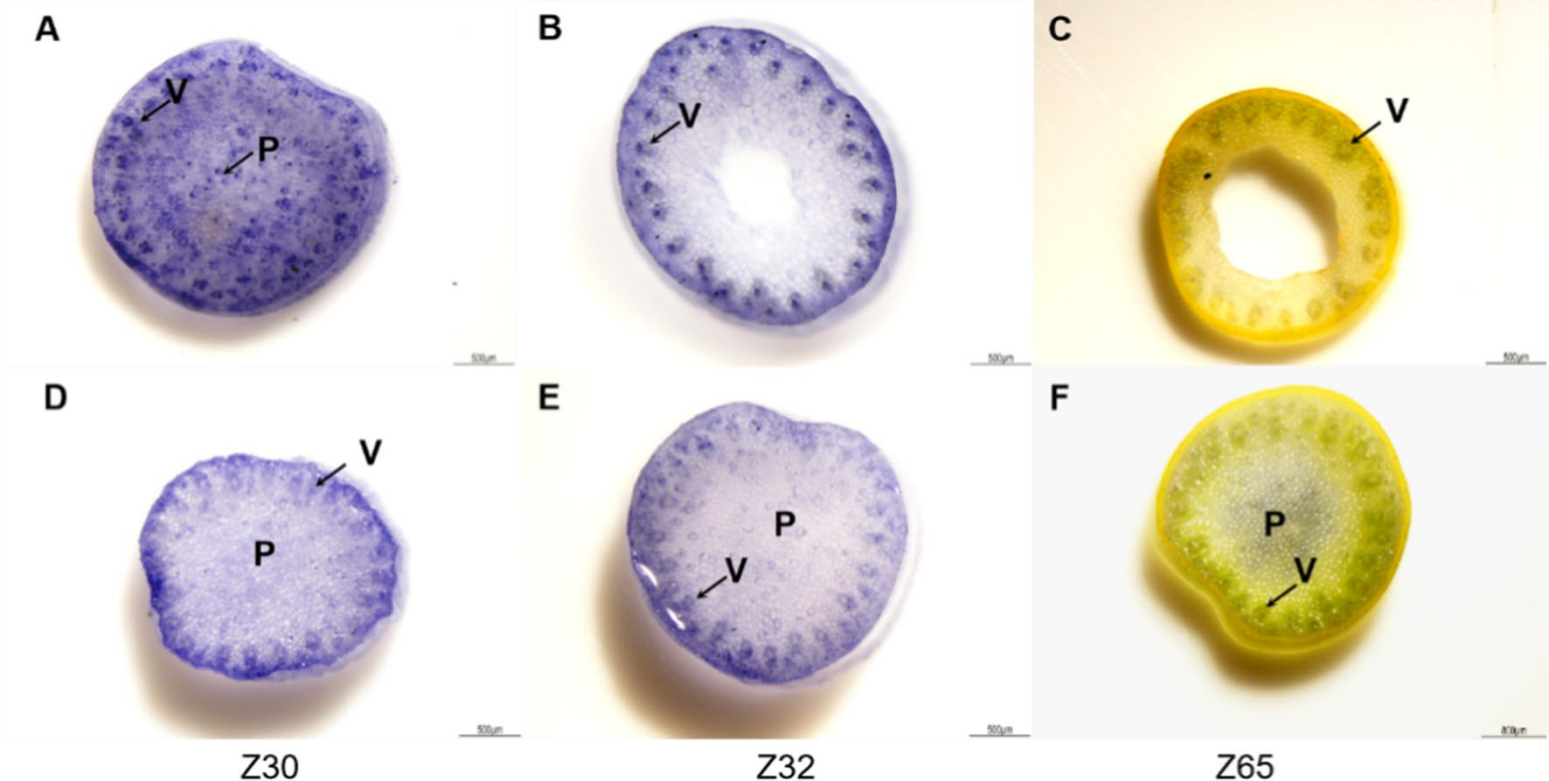
NBT staining for ROS in wheat stems at Z30, Z32 and Z65 stage. NBT reacts with ROS to form a dark blue insoluble formazan compound. ‘Kauz’ stem section **A**–**C**, ‘Westonia’ stem section (**D–F**). V, xylem vessel elements; P, pith parenchyma cells. The staining differences are mark by arrows (colour figure online)

As reflected by the GO and KEGG analyses, significantly enriched DEGs were found related to oxidative stress and ROS scavenging pathway in low PT samples. We suspected that ROS metabolism of which might be more active than that in high PT samples. This was consistent with the ROS accumulation in stems detected by NBT staining. At Z30 stage, the dark blue deposit was found in the pith cells and xylem vessel elements in the stem of ‘Kauz’ (Fig. 4A), but no obvious staining was found in ‘Westonia’ (Fig. 4D). At Z32 stage, only xylem vessel elements of ‘Kauz’ were intensely stained by NBT (Fig. 4B), while, at Z65 stage, neither ‘Kauz’ nor ‘Westonia’ showed strong staining signals (Fig. 4C, D). This result confirmed the accumulation of ROS in ‘Kauz’ was higher than that in ‘Westonia’, and the active ROS metabolism in ‘Kauz’ may be involved in pith PCD, as ROS can induce cell death.

### *SNP calling and DEG discovery *via* BSR-seq*

**Fig. 5:**
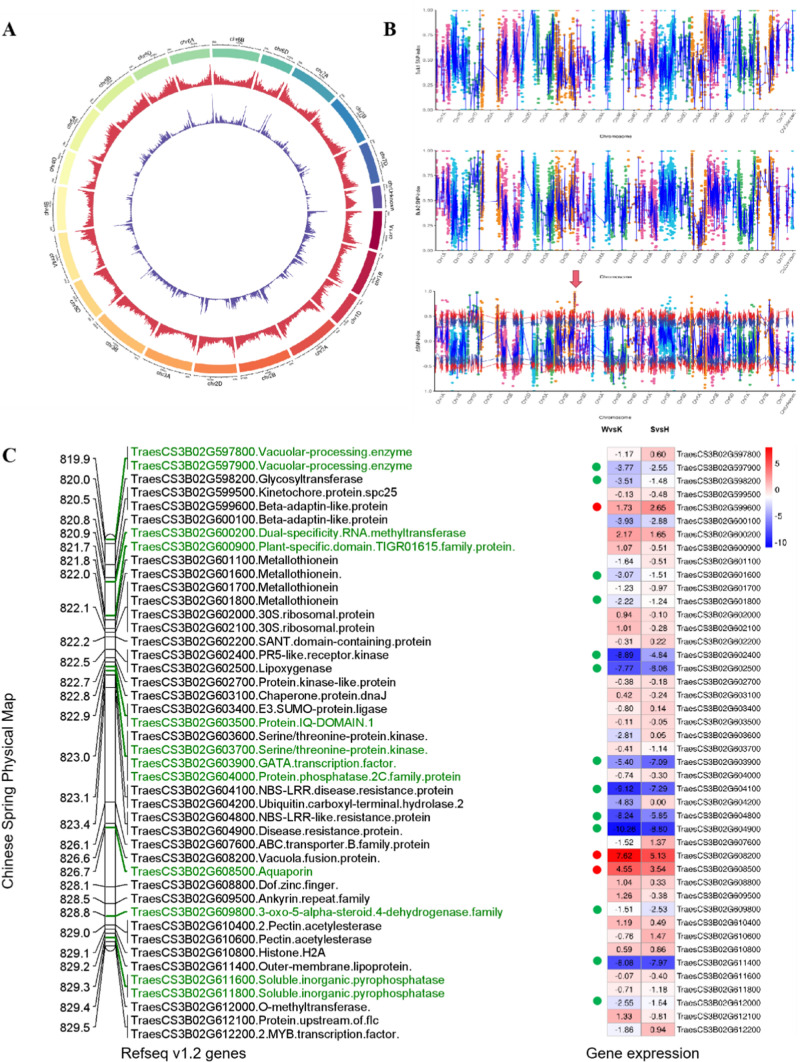
SNP calling and DEG discovery via BSR-Seq. **A** Circos graph of genome-wide genes and SNPs distribution. The outer circle represents chromosomes, the middle circle represents gene distribution, and the inner circle represents the SNP density distribution. **B**: BSR-seq mapping of pith-thickness based on ∆SNP index value. ‘Bulk1’ represent the Hbulk SNP index, ‘Bulk2’ represent the Sbulk SNP index. The x-axis represents the position of chromosomes and the y-axis represents the SNP index or ∆SNP index value. The blue line represents the average value of ∆SNP index which was computed in a 5 Mb interval using a 50 kb sliding window. The red line and purple line represent the 99% and 95% confidence level threshold, respectively. **C** Gene expression comparison within the pith-thickness interval in the Chinese spring chromosome 3B as the reference. Physical positions are shown to the left of the map in Mb. Genes that contained SNPs between two parents are highlighted in green font. Gene expression differences between ‘Westonia vs Kauz’ and ‘Sbulk vs Hbulk’ comparisons are shown as a heatmap on the right. Positive fold changes shown in red shading indicate higher expression in the solid sample. Negative fold changes shown in blue shading indicate higher expression in the hollow sample. Expression values are expressed as log2 FC. The red dot highlights the DEGs with upregulated expression in both ‘Westonia’ and solid bulk; the green dot highlights DEGs with downregulated expression in both ‘Kauz’ and hollow bulk (colour figure online)

We further identified 72,301 expressed genes from four sequencing libraries and 352,388 SNPs were called through GATK in total. The genome-wide SNPs distribution was shown in Fig. 5A. After filtering, 11,331 high-quality SNPs were obtained (Table S5). The average density of SNPs on all chromosomes was 0.74 SNPs per Mb, with the highest density on chromosome 5B (1.47 SNPs per Mb) and the lowest density on chromosome 4D (0.11 SNPs per Mb) (Table S5). The sequencing depth of the four samples ranged from 6.84 × to 9.93 × ; 2275 of the SNPs were nonsynonymous. Under the threshold of 99% confidence, only one putative candidate region has been revealed (Table S6, Fig. 5B). This region contains the *Qpt-3B* QTL with a genomic size of 6.83 Mb (819, 897, 386–826, 725, 912 bp).

Using ∆SNP > 0.74 as a threshold (Hao et al. 2019), a total of 25 SNPs with high confidence were identified in this region, and 24 SNPs were in the exon region (Table S7). The variants in the *Qpt-3B* QTL region were examined using the Integrative Genomics Viewer (IGV) (Thorvaldsdóttir et al. 2013). One gene (*TraesCS3B02G597900*) showed consistent frequencies of DNA variants with corresponding parents at about 0% in Hbulks and 100% in Sbulks (Fig. S2).

By searching the candidate region and adjacent region, a total of 143 high confidence genes were included, among which 44 genes were detected in at least one comparison group through RNA-seq. The expression analysis revealed that all these expressed genes could be divided into four categories. The first two categories include the genes only expressed in one of the samples. In the other two categories, genes are expressed in both samples but up-regulated in one of the samples (Table S8 and Table S9). We found 16 coexisting DEGs with the same expression pattern in two comparison groups. Among them, 12 DEGs were highly expressed (> twofold) in both ‘Kauz’ and Hbulk, while 4 DEGs (*TraesCS3B02G597800, TraesCS3B02G597900, TraesCS3B02G603900* and *TraesCS3B02G608500*) were highly expressed in both ‘Westonia’ and Sbulk (Fig. 5C). These four genes have potentially deleterious SNPs and Indels related to high PT phenotype (Table S7). The expression levels of *TraesCS3B02G597800, TraesCS3B02G597900 and TraesCS3B02G603900* were higher in low PT samples ‘Kauz’ and ‘Hbulk’, while *TraesCS3B02G608500* was higher in high PT samples ‘Westonia’ and ‘Sbulk’.

### qRT-PCR

**Fig. 6:**
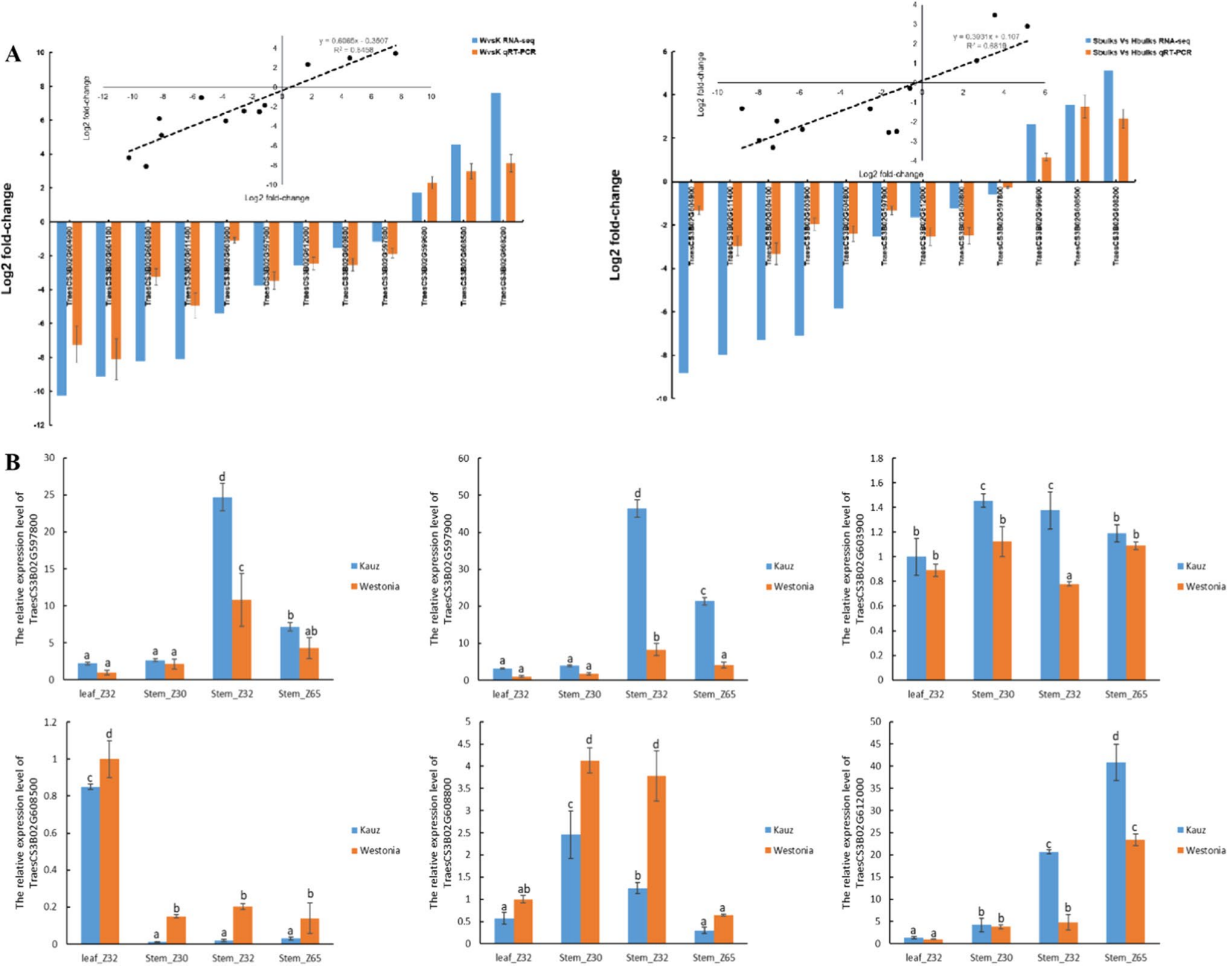
Real-time quantitative PCR of candidate genes at three development stage in two parental lines. **A** Validation of RNA-seq data for differential gene expression by qRT-PCR. Inset: simple correlation plot of the log2 FC in expression obtained by RNA-Seq (x-axis) and qRT-PCR (y-axis). **B** Expression profiles of two VPEs, Dof, GATA, COMT and Aquaporin in each corresponding period of two parents in leaves and stems, respectively (colour figure online)

Twelve DEGs in the 3BL candidate region were validated through real-time qRT-PCR to verify the authenticity of RNA-seq results. The designed primer set was listed (Table S3). The qRT-PCR results confirmed the direction of regulation (positive or negative) between high and low PT samples for selected genes. The log_2_ fold-change (log_2_FC) value was also similar for the majority of genes, with a correlation coefficient of 0.85 and 0.68 between RNA-seq and qRT-PCR data sets derived from ‘Westonia vs Kauz’ and ‘Sbulks vs Hbulks’, respectively (Fig. 6A).

In addition, six genes were selected for further investigation of gene expression profiles at three stem developmental stages in two different tissues (Fig. 6B). Among them, four DEGs with selected based on SNPs, while the other two DEGs were selected based on gene annotation results. *TraesCS3B02G608800* was annotated as a *Dof* transcription factor, which might be involved in regulating stem pith cell apoptosis. *TraesCS3B02G612000* was annotated as Caffeic acid 3-O-methyltransferase (*COMT*), which might be involved in the stem lignin synthesis pathway.

Except for the GATA transcription factor gene (*TraesCS3B02G603900*) and the aquaporin gene (*TraesCS3B02G608500*)*,* the other four genes showed significant expression differences at three developmental stages. The expression profiles of two *VPE* genes (*TraesCS3B02G597800 and TraesCS3B02G597900*) were similar, displaying high expressions in stems than in leaves. Their expression levels were significantly higher in low PT ‘Kauz’, with the highest transcript abundance at Z32 stage then showed a downward trend at Z65 stage. *COMT* gene (*TraesCS3B02G612000*) also maintained a higher relative expression level in ‘Kauz’, with the gene transcript abundance increasing gradually and reaching peak at the flowering stage.

In addition, the *Dof* gene (*TraesCS3B02G608800*) was highly expressed in ‘Westonia’ and the transcript abundance was the highest at the early stage of stem tissue in both parents and then gradually decreased during development. It can be seen that the expression levels of *VPE*, *DOF* and *COMT* genes varied between developing stages. *VPE* and *DOF* were highly expressed at the stem elongation stage (Z32) when the stem pith cells were undergoing autolysis; while *COMT* accumulated significantly at the flowering stage (Z65). Considering that VPE is a cysteine-type endopeptidase and plays an important role in regulating the programmed death of plant cells, we concluded that *TraesCS3B02G597800 and TraesCS3B02G597900* are more likely to be the genes responsible for the phenotypic differences in pith thickness.

### Sequencing and phylogenetic analysis of TraesCS3B02G597900

In the candidate region, the CDS of six genes were amplified in the parents. Primers for six genes (*TraesCS3B02G597800, TraesCS3B02G597900, TraesCS3B02G603900*, *TraesCS3B02G608500, TraesCS3B02G608800* and *TraesCS3B02G612000*) are shown in the Table S3. The corresponding PCR products were sequenced and aligned with the reference genome (IWGSC v2.1).

**Fig. 7:**
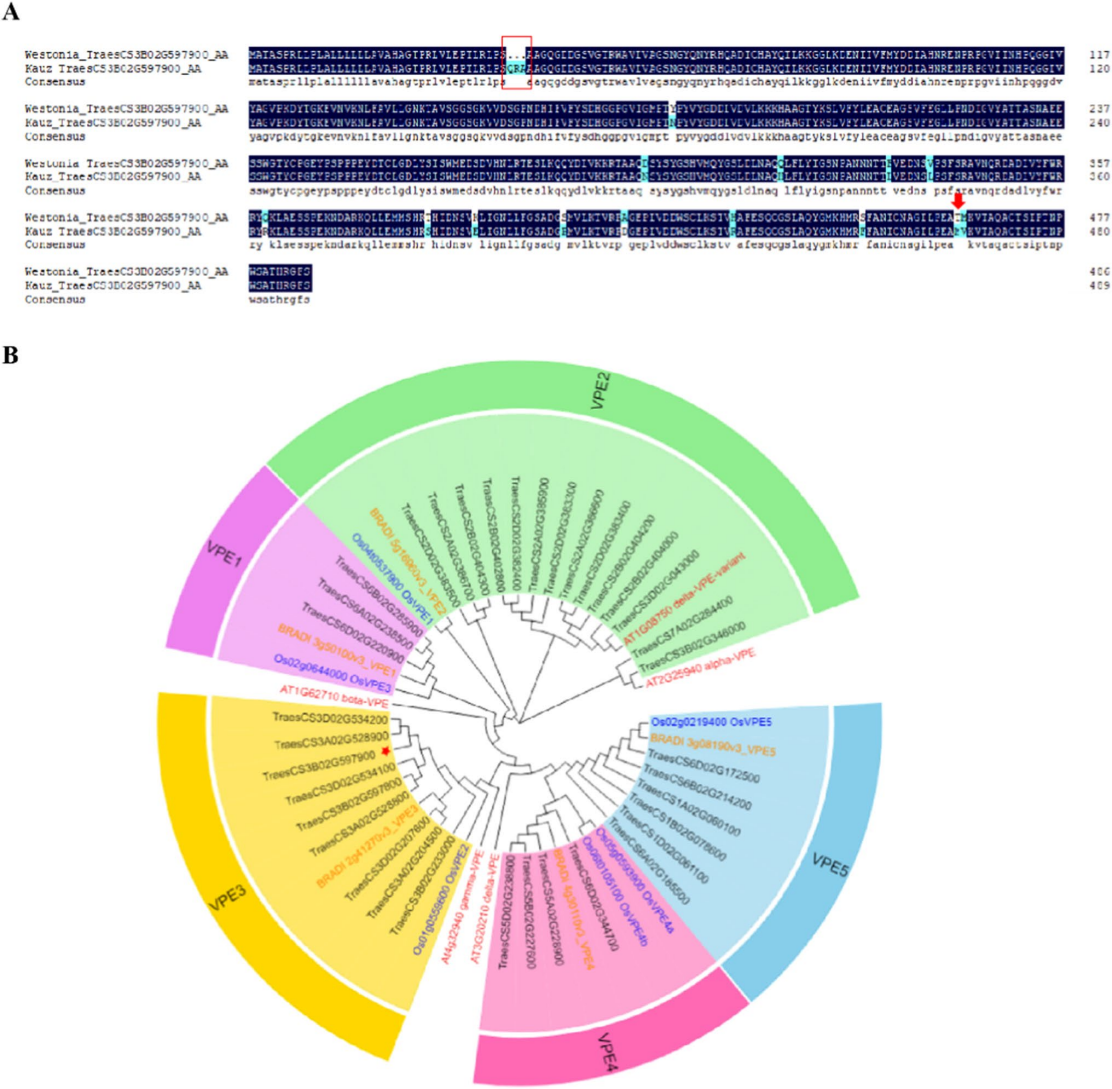
Amino acid sequence alignment and phylogenetic tree of *TraesCS3B02G597900*. **A** Alignment of the deduced amino acid sequences of *TraesCS3B02G597900* from ‘Westonia’ and ‘Kauz’. The red rectangle represents a 9 bp Indel. The red triangle represents an amino acid substitution M465T with SIFT = 0.01; **B** Phylogenetic tree of vacuolar processing enzyme family. This tree illustrates the VPE1-5 family groups and includes VPE proteins from *Arabidopsis* (red font), rice (blue font), *Brachypodium* (orange font) and wheat (black font). Red star represents *TraesCS3B02G597900* (colour figure online)

*TraesCS3B02G597800* and *TraesCS3B02G597900* from low PT stem parent ‘Kauz’ shared the same sequence as the reference. The sequence of *TraesCS3B02G597800* in high PT parent ‘Westonia’ contains only one missense SNP, while *TraesCS3B02G597900* has not only several point mutations but also a 9-bp deletion in the first exon, resulting in a 3-aa deletion and 14 amino acid substitutions in ‘Westonia’ (Fig. 7A, Fig. S3). Of the 14 amino acid substitutions, M465T displayed an extremely low SIFT (Sorting Intolerant from Tolerant) score of 0.01, implying that the substitution could affect the protein function according to Sim et al. (2012).

Phylogenetic analysis was performed on putative VPE amino acid sequences from common wheat, *Brachypodium distachyon* (a relative of the wheat), the distant relative rice (*Oryza sativa*) and model plant *Arabidopsis thaliana*. The phylogenetic tree showed that wheat VPEs can be clustered into five clades with one *Brachypodium* VPE in each clade, including the endosperm-specific VPE1, the pericarp-specific VPE4, and vegetative tissue-specific VPE3 and VPE5. For the VPE3 subfamilies, the wheat genome harbours three copies (VPE3a, 3b and 3c) with one from each of the three sub-genomes. *TraesCS3B02G597900* belongs to VPE3 family (Fig. 7B). Therefore, we named it *TaVPE3cB*.

*TaVPE3* contains two conserved domains, peptidase C13 domain and legumain C domain. The N-terminal catalytic domain is a caspase-like from C13 family and the C-terminal is involved in legumain stabilization and activity modulation (LSAM). In high PT parent ‘Westonia’, *TaVPE3cB* contains three amino acid substitutions in the catalytic domain, one in the activation peptide and eight in the LSAM domain (Fig. S4). Nevertheless, there was no substitution of the key amino acids in the substrate pocket and catalytic dyad site (Hara-Nishimura et al. 2005).

The sequence similarity analysis in ten common wheat varieties (‘Westonia’, ‘Lancer’, ‘CDC Landmark’, ‘Claire’, ‘Janz’, ‘Chinese Spring’, ‘Julius’, ‘SyMattis’, ‘Weebill’, ‘Kauz’) revealed that *TaVPE3cB* has two natural allelic variations. High pith-thickness cultivars, such as ‘Lancer’, ‘CDC Landmark’ and ‘Janz’, shared the same allele as ‘Westonia’, which was named *TaVPE3cB.b*; while low pith-thickness ‘Kauz’ carries the same allele as ‘Chinese spring’, ‘Julius’, ‘SyMattis’ and ‘Weebill’, which was named *TaVPE3cB*.a (Fig. S4).

Next, we cloned the 1 kb promoter of *TaVPE3cB*.a and *TaVPE3cB.b*. Sequence alignment revealed 73.83% sequence identity and a 309 bp insertion at 284 bp upstream of the start codon in the promoter of *TaVPE3cB.b* in Westonia (Fig. S5). We also carried out predictive analysis of the cis-acting elements in the promoters of *TaVPE3cB.a* and *TaVPE3cB.b* using PlantCARE. The analysis revealed various possible cis-acting elements in the two promoters that were mostly related to phytohormone response and stress induction, implying that *TaVPE3cB* may participate in the regulation of multiple phytohormones and environmental signalling pathways. Within the 309 bp insertion in the promoter of *TaVPE3cB.b*, 21 cis-acting elements exist including one unique cis-acting element MBS (MYB binding site involved in drought-inducibility, CAACTG) motif.

### SNP marker development and linkage analysis for a major pith-thickness locus on 3BL

To facilitate the use of *TaVPE3cB.b* in wheat-breeding programs and confirm that all high pith-thickness accessions contain *TaVPE3cB.b* allele with the 309-bp insertion in the promoter, we developed two allele-specific PCR markers (Table S3), *Qpt3B-F1/R1* (dominant SNP marker) and *Qpt3B-F2/R2* (codominant Indel marker for the promoter). The PCR products of *Qpt3B-F1/R1* were 1097 bp in size for the varieties carrying *TaVPE3cB.b* allele, whereas no bands were amplified for the varieties carrying *TaVPE3cB.a*. The PCR products of *Qpt3B-F2/R2* displayed a 634-bp band from *TaVPE3cB.b*, whereas a 325 bp band was observed in the varieties containing *TaVPE3cB.a* (Fig. S6). The genotyping results obtained from these two pairs of markers were the same.

**Fig. 8:**
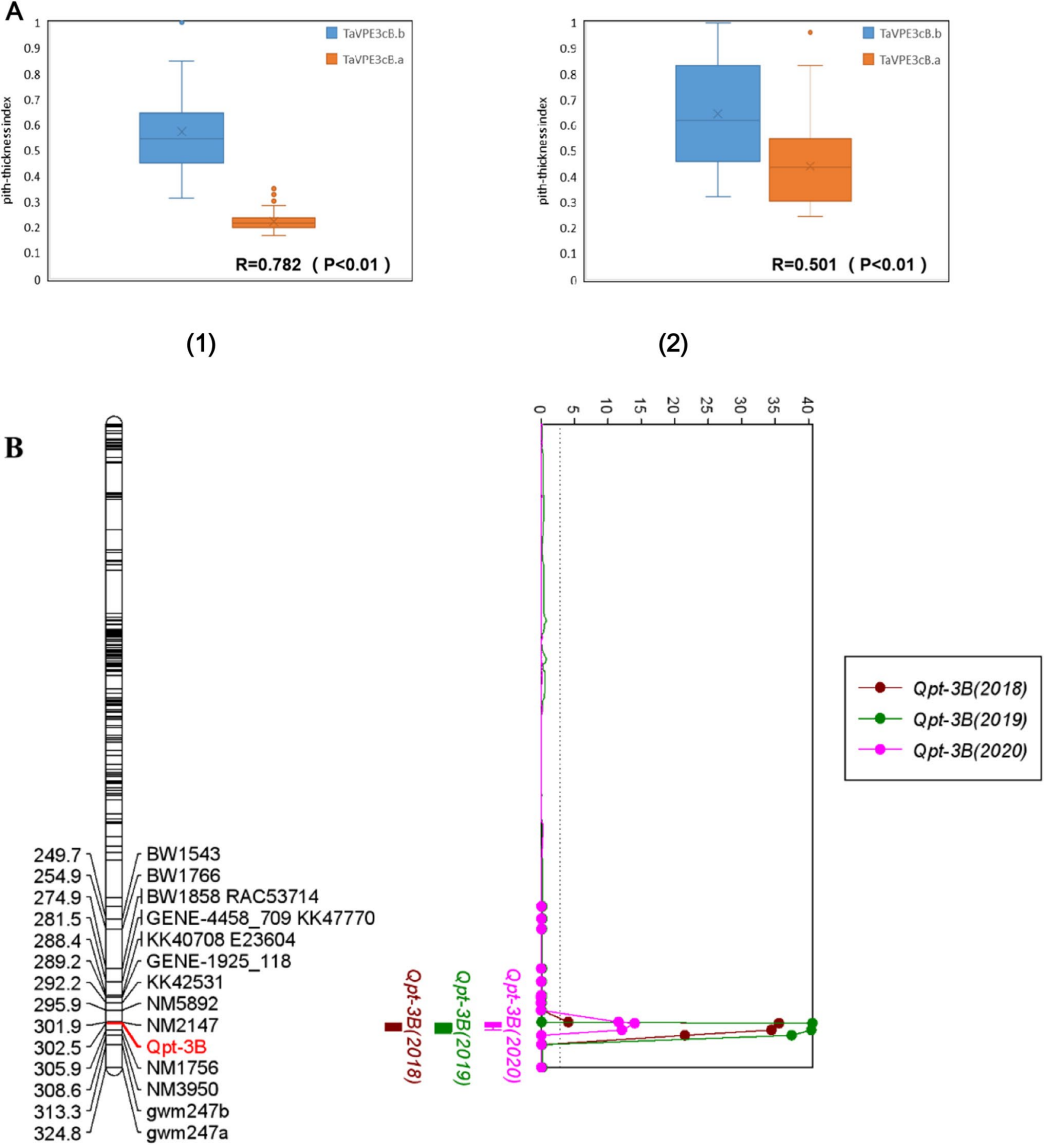
*TaVPE3cB* marker development and linkage map analysis. **A** Pith-thickness index in *TaVPE3cB.a* and *TaVPE3cB.b* genotypes of DH population (**a**) and historical lines (**b**). **B** Location of QTL for PT on 3B under three individual environments (2018-2020)

Using these two pairs of allele-specific PCR markers to screen the DH population and historical varieties, we found that the *TaVPE3cB.b* genotype is closely linked to the high PT phenotype, while *TaVPE3cB.a* was associated with the low pith-thickness phenotype (Fig. 8A). The discrimination rate of this marker in high PT (PI > 0.6) DH lines was 100%, and 87.64% in low PT DH lines (PI < 0.4). A Spearman’s correlation coefficient of 0.782 (*P* < 0.01) between the PT index and *TaVPE3cB.b* gene demonstrated that *TaVPE3cB.b* significantly increase wheat stem pith-thickness in the DH population. However, the marker discrimination rate was low to 74.41% in high pith-thickness historical varieties (PI > 0.6), demonstrating the presence of other PT-related loci in some wheat varieties. When extreme phenotype varieties with solid stem (PI > 0.8) were tested, the detection rate was 89.47%. The discrimination rate of low PT (PI < 0.4) varieties was 89.79%, which was consistent with that in low PT DH lines, and the Spearman’s correlation coefficient was 0.501 (*P* < 0.01) between the PT index and *TaVPE3cB.b* gene. Therefore, this marker can be useful to identify the allele of *QTL-3B* for wheat varieties.

The newly developed makers of *Qpt3B* were integrated into the previous QTL-3B linkage map developed by Zhao (2019). Finally, this marker was mapped in the genetic region of 302.5 cM. The QTL linked to high pith-thickness was detected under three individual environments using DH lines. This QTL was confined to an interval of 3.41 cM flanked by markers *Qpt3B* and *NM3950* (Fig. 8B) and explained 68.72% and 13.85% of the phenotypic variance with the LOD value of 40.59 and 12.13, respectively.

### Expression analysis of TaVPE3cB

**Fig. 9:**
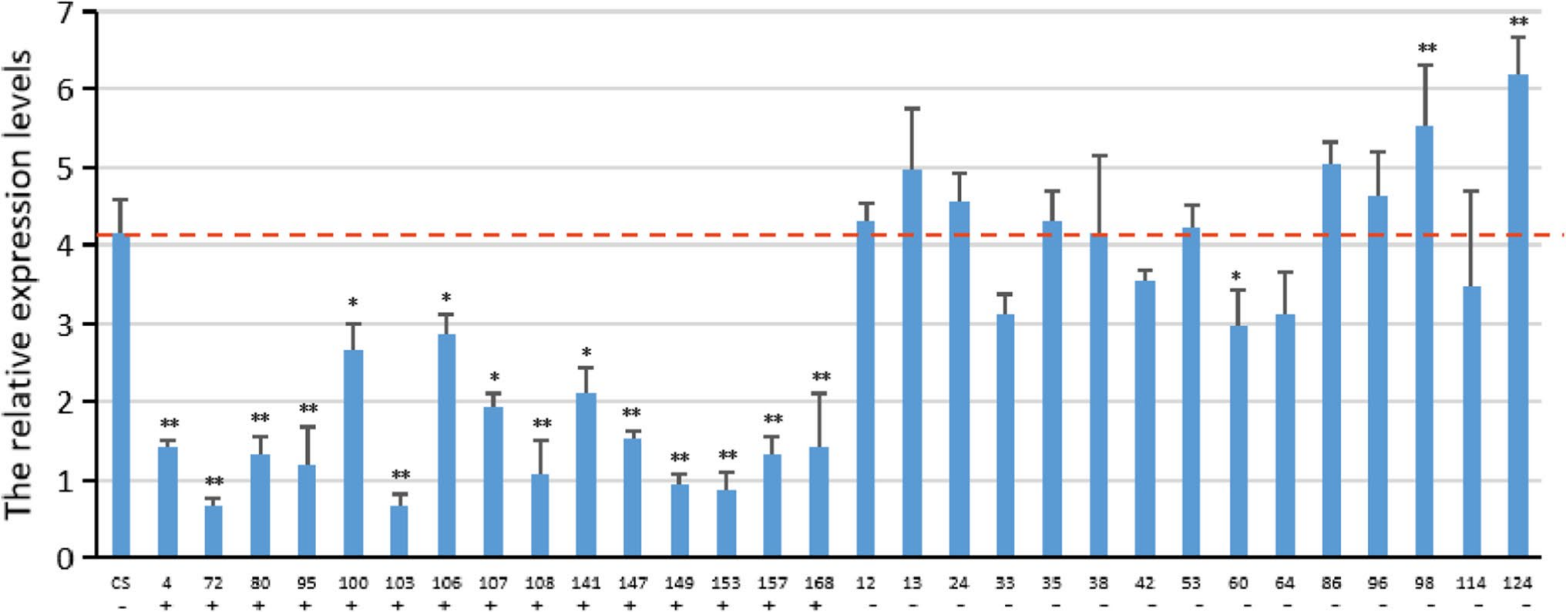
Expression analysis of *TaVPE3cB.a* and *TaVPE3cB.b* in historical wheat varieties. The red line shows the relative expression levels of *TaVPE3cB* in CS which contains *TaVPE3cB.a* (control). + , − Denote *TaVPE3cB.b* containing the 309 bp insertion and *TaVPE3cB.a* missing the 309 bp insertion in the promoter, respectively. ∗, ∗∗Denotes significant differences at 5% and 1% probability levels, respectively (colour figure online)

Promoter structural analysis has wide implications in the prediction of gene expression profiles. We analyzed the GC-content in the 1.5 kb fragment upstream from the translation initiation codon of the *TaVPE3cB* allelic variations and found that the AT-content of *TaVPE3cB.a* and *TaVPE3cB.b* promoter was 56% and 53%, respectively, higher than the corresponding GC content, which is characteristic of an AT-rich plant gene-promoter element. To clarify the contribution of the 309 bp indel to gene expression, we performed qRT-PCR using the stems of ‘Chinese spring’ (CS) and 15 varieties containing *TaVPE3cB.a* genotype and 15 varieties containing *TaVPE3cB.b* genotype, which were selected from 171 historical varieties. The expression in all varieties containing *TaVPE3cB.b* (with 309 bp insertion) was lower than that in those containing *TaVPE3cB.a* (without the 309 bp insertion) (Fig. 9). This indicates that this 309 bp insertion can downregulate the expression of *TaVPE3cB.b*, which further inhibits pith death to induce a high PT stem phenotype.

## Discussion

### Insights of pith thickness formation mechanism

Wheat stems are generally solid in the nodal region at the initial developmental stage, followed by an internodal cavity formation due to the death of pith cells during internode elongation. The genes responsible for stem pith-thickness are probably involved in the death of pith cells and cell wall composition. For example, pith thickness can be modulated by activating or inhibiting PCD (Fujimoto et al. 2018), or changing the cell wall composition, increasing stem cell wall thickness and lignin content (Kong et al. 2013).

### Plant cysteine proteases and PCD of stem

Previous studies have revealed the roles of cysteine proteases in plant development as PCD initiators and executors (Rustgi et al. 2017; Sueldo and van der Hoorn 2017; Zhang et al. 2014). VPEs are cysteine proteinases and have important functions in the processing and maturation of proteins and PCD in the plant (Hara-Nishimura et al. 1993). Four functional VPE isoforms (α, β, γ, and δ-VPE) have been identified in *Arabidopsis* (Shimada et al. 2003). They can be divided into two subfamilies: seed type (β and δ- VPE) and vegetative type (α and γ- VPE), which are expressed primarily in seeds and vegetative organs, respectively. Seed type VPEs involve in the processing and maturation of seed storage proteins (Gruis et al. 2004), while vegetative type VPEs are found in lytic vacuoles and have been confirmed to involve in plant PCD and may act as functional substitution of caspases (Hatsugai et al. 2015). Arabidopsis γvpe mutants revealed that hypersensitive response related to PCD is reduced and the susceptibility to pathogens is increased in the absence of γVPE, as cell death can be blocked (Rojo et al. 2004).

In this study, the GO enrichment analysis revealed that the Hcluster contains 35 up-regulated genes encoding for enzymes with aspartic, serine, and cysteine endopeptidase activity. We also found that *TaVPE3cB* was highly expressed in stem tissues rather than leaves at the elongation stage. The results are consistent with the finding of Kinoshita et al. (1995) in that *γVPE* is expressed predominantly in the stem of wheat. Recently, Cheng et al. (2019) demonstrated that *γVPE* regulates xylem fiber cell PCD by activating cysteine endopeptidase 1 (*CEP1*) during stem development, and *CEP1* can function as an executor in clearing cellular contents during PCD in xylem development. Moreover, the mutation of γVPE exhibited a similar phenotype as *cep1* mutant, such as incomplete degradation of the cellular contents and thickening secondary cell walls, which was caused by the prolonged PCD in xylem cells (Han et al. 2019). It was concluded that *γ*VPE is not only involved in the maturation of CEP1, and also plays an important role in regulating the degradation of cellular content and the thickening of the secondary cell wall (Cheng et al. 2019). And more notably, Fujimoto et al. (2018) identified a NAC transcription factor and referred as *D* gene (*Sobic.006G147400)*, which up-regulated the expression of *CEP1* and *VPEs*, thus triggering pith parenchyma cell PCD in sorghum. Therefore, we can speculate that the downregulated expression of *TaVPE3cB* blocked pith cell apoptosis, leading to thicker pith tissue.

### Transcriptional regulatory factors and PCD of stem

A putative Dof zinc finger protein (*TraesCS3B02G608800*) was found in the adjacent region of PT QTL. It is the ortholog gene of *TdDof* (*TRITD3Bv1G280530*). Nilsen et al. (2020) demonstrated that multiple copy numbers of *TdDof* increased the accumulation of gene transcripts and eventually inhibited PCD in pith parenchyma cells of solid-stemmed durum wheat. However, an exception was found in an Australia common wheat cultivar ‘Janz’, which only had a single copy of *Dof* but still exhibited the solid stemmed phenotype (Beres et al. 2013; Nilsen 2017). In addition, the relative copy number of *TaDof* gene was estimated with the ∆∆CT method using a single copy gene, *TraesCS3B02G612200,* as the endogenous control gene. No copy number difference was found between the two parental lines and among the DH lines. In addition, the gene fell outside of our defined QTL interval, suggesting that it is not a strong candidate gene of the QTL identified in the ‘Westonia’/ ‘Kauz’ DH population.

In the current study, the PT QTL interval also harbours a GATA 17 transaction factor gene (*TraesCS3B02G603900*). GATA 12 has been reported to be involved in regulating the processes of plant xylem vessel differentiation and PCD (Cubría-Radío and Nowack 2019). For example, overexpression of *AtGATA12* in *Arabidopsis* can induce the formation of ectopic xylem vessel-like elements by manipulating the expression of VND7 transcription factor (Endo et al. 2015). Similarly, overexpression of *PtrGATA12* in poplar resulted in increased contents of lignin and secondary cell wall (SCW) thickness by controlling the expressions of some master TFs and pathway genes involved in SCW formation and PCD. Moreover, the *PtrGATA12* transgenic lines exhibited significantly increased stem diameter (Ren et al. 2021). Besides, *GATA19* has been proved to be involved in regulating plant growth rate. For instance, *PdGATA19* transgenic lines exhibited increased biomass accumulation, stem height and photosynthetic rate; while CRISPR/Cas9-mediated mutant plants showed severe developmental retardation and increased formation of secondary xylem (An et al. 2020). In this study, three polymorphic SNPs with higher ΔSNP index values (> 0.75) were found in this gene. Both RNA-seq and qRT-PCR showed that the expression level of this gene is also different between the two parents as well as the two bulked samples.

### Cell wall modification and cell expansion

*COMT* is considered as an important gene that functions in lignin biosynthesis, and it is positively correlated with lignin content in wheat stems (Bi et al. 2011). Lignin deposition could reinforce the cell wall to provide mechanical support to the stem which makes it possible to modify stem strength and lodging resistance by affecting lignin content (Ma 2009; Tu et al. 2010).

A putative O-methyltransferase gene (*TraesCS3B02G612000)* which contains the *O-MeTrfase_COMT* domain was located on the adjacent region of the target QTL. This gene was suggested as a promising candidate gene for stem pith production based on strong differential expression between solid and hollow cultivars (Oiestad et al. 2017). In the current study, no SNP for *COMT* was found between the two parental lines. However, it was significantly up-regulated in low PT ‘Kauz’ and hollow bulked samples with log_2_ FC of 2.25 and 1.64, respectively. In addition, GO analysis also identified significant functional enrichment of O-methyltransferase in Hcluster. This suggests that the activity of cellular lignification by *COMT* was lower in high PT Wheat, which is consistent with the result reported by Nilsen (2017). Taking together, *TraesCS3B02G612000* affects PT is probably through lignifying the stem pith cell wall.

An aquaporin gene (*TraesCS3B02G608500*) was also observed adjacent to the PT QTL interval. Aquaporins are universal membrane integrated water channel proteins which play an important role in cell expansion and cell division by controlling water uptake (Maurel et al. 2015). Fujimoto et al. (2018) found high PT stems were filled with plump pith cells which enhanced stem water content. In this study, a tonoplast intrinsic protein aquaporin gene (*TIP1*) with two nonsynonymous substitutions in the coding region showed significant expression-level differences. It was significantly upregulated in high PT samples but with low expression abundance (FPKM < 0.8). Previous studies showed a correlation between *TIP1* expression level with cell elongation and differentiation in the vascular tissue of *Arabidopsis thaliana* (Ludevid et al. 1992). In addition to *TIP*, the plasma membrane intrinsic aquaporin (*PIP*) has been implicated in plant stem growth. For example, increasing the expression of *PIP1b* in transgenic tobacco can improve water transport and transgenic plants with thicker stem diameters more than those of wild-type plants (Aharon et al. 2003). Yu et al. (2005) found that *PIP1* antisense transgenic tobacco plants displayed thicker and shorter stems than wild-type plants. Therefore, the differential expression of aquaporin genes may be the cause of differences in pith cell expansion and water uptake between the two stem phenotypes.

### Metallothionein and PCD of stem

**Fig. 10:**
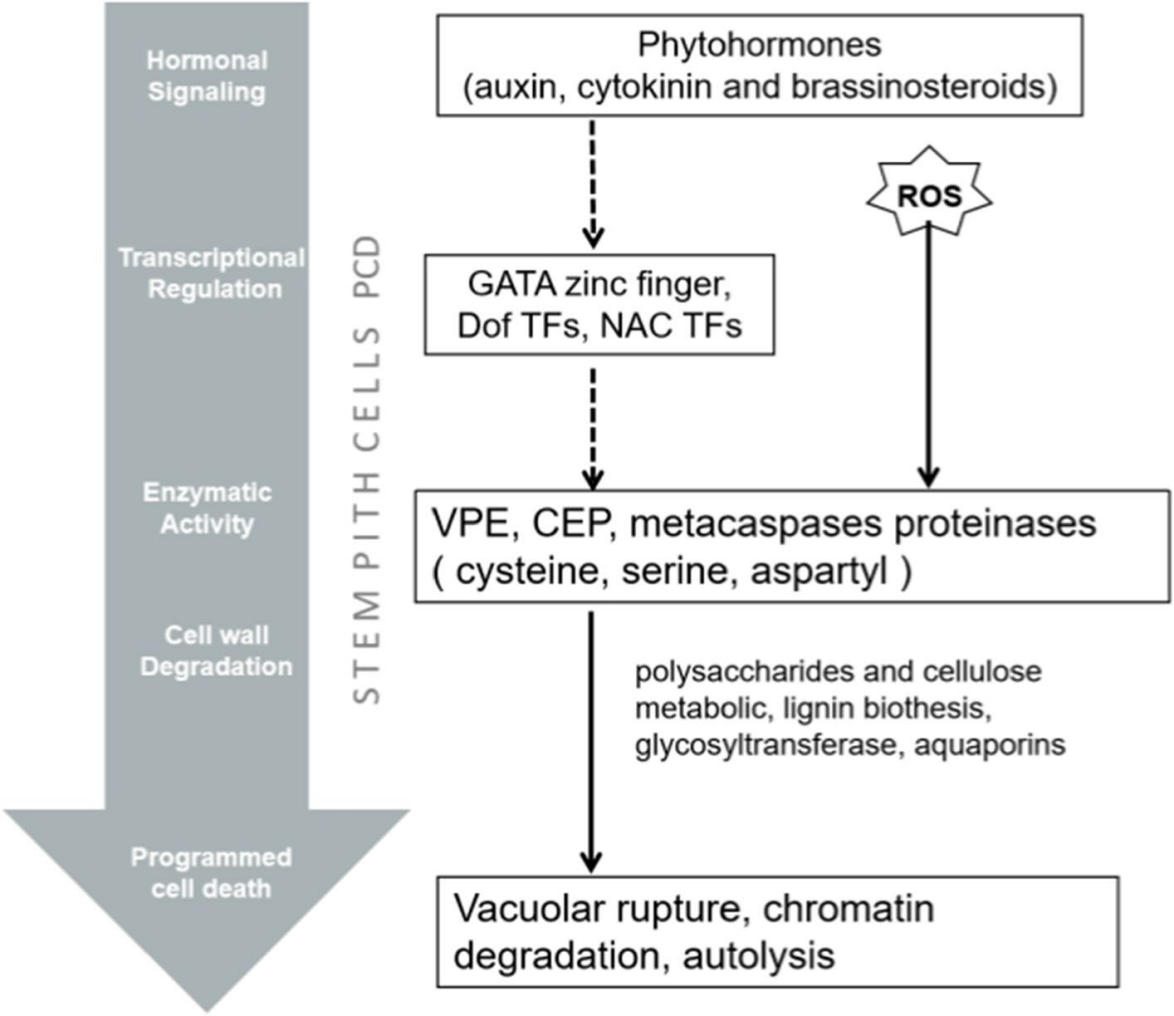
Five-level hierarchy diagram of stem pith cells PCD. Dotted lines indicated indirect regulation; solid lines indicate direct regulation

Plant metallothionein (MT) is a small and functionally, cysteine-rich protein that plays multiple roles in reactive oxygen species (ROS) scavenging. Metallothionein protein functions as a cytosol ROS scavenger, it can stall the signal transduction of ROS-mediated PCD, which is a widespread regulatory mechanism for eliminating unwanted cells in normal plant growth and development. For example, the *OsMT2b* gene in rice and the *MT3a* gene in cotton exhibited strong antioxidative activities against ROS (Xue et al. 2009). Knocking out *OsMT2b* caused excessive epidermal cell death in stems (Steffens and Sauter 2009). Beers (1997) proposed that PCD is essential for eliminating pith parenchyma cells and forming aerenchyma to facilitate gas exchange. In addition, previous studies have shown that pith PCD activation is inhibited in solid stemmed wheat during stem elongation (Nilsen et al. 2020). Therefore, MT could be involved in the normal PCD of pith cells. In this study, we observed significant up-regulation of *MT* genes in low PT samples according to RNA-seq results, which may be due to the antioxidant defence mechanism increasing the expression of ROS scavenger genes, thus mitigating the damage caused by ROS. Based on the above results, we proposed a regulation model for the formation of wheat hollow stems (Fig. 10). The topmost level of regulation of the differences between high PT and low PT stem was correlated with a hormonal signalling pathway. Many DEGs involved in auxin, cytokine and brassinosteroid plant hormone signal transduction were revealed by KEGG analysis, and the role of this pathway in regulating stem elongation has been reviewed by Haruta and Sussman (2017). In the downstream of hormonal signalling, multiple transcription factors such as *NAC*, *Dof* and *GATA* have been involved in cell differentiation including PCD as its final destination. In this pathway, numerous proteases participate in PCD execution. The most common executor is cysteine proteases, such as CEP1, VPE and metacaspases, which have been shown to contribute to cellular autolysis before and after PCD. In addition, ROS can act as the cell death signal in the MAPK signalling pathway together with hormones to activate protease to initiate cell death (Biswas and Mano 2016; Li et al. 2012; Overmyer et al. 2003). After hydrolytic enzymes are released, the cell wall breakdown and cell wall components recombination occurs, as many DEGs are related to polysaccharides and cellulose metabolic, lignin biosynthesis, glycosyltransferase and aquaporins, which can modify the cell wall component (Gunawardena et al. 2007). Eventually, the pith cell vacuolar ruptures and triggers chromatin degradation (Obara et al. 2001).

### Comparison of genes/QTLs for stem-related traits

In the wheat breeding program (Dreccer et al. 2020), the design of wheat varieties with wider stem diameter, high culm wall thickness, small pith cavity and high stem solidness is desirable for enhancing stem strength and lodging resistance. Using forward genetic approaches, several strong stem phenotype-related QTLs have been identified. *SSt1* in durum wheat and *Qss.msub-3BL* in common wheat are the earliest mapped stem solidness loci on chromosome 3B. Later, loci on 1A, 2D, 3B and 4B for culm wall thickness and pith diameter were identified. However, only the loci on 1A for culm wall thickness and the 3B locus for stem solidness have been identified through map-based cloning. The *Csl* is the candidate gene from chromosome 1A, which altered carbon partitioning throughout the plant and increased the cell wall thickness (Hyles et al. 2017). *TdDof* (*TRITD3Bv1G280530*) was cloned as the most likely *SSt1* candidate gene due to different copy numbers in solid-stemmed and hollow-stemmed durum wheat lines. Likewise, *TaDof* (*TraesCS3B01G608800*) has been reported as the candidate gene for *Qss.msub-3BL*.

In previous studies of our group, Zhao (2019) detected the major QTLs for wheat pith thickness and stem diameter on 3BL by using DH population from ‘Westonia’/ ‘Kauz’. The 3B QTL for PT was saturated to a 3.0 cM interval which corresponded to a 1.43 Mb (820,760,675–822,192,510 bp) physical region and was stably expressed in five different environments. In this study, one PT-related candidate interval was identified at a 6.83 Mb physical interval (819,897,386–826,725,912 bp) of 3BL using BSR-seq data. However, the reported *TaDof* in *Qss.msub-3BL* is situated at 828,110,748–828,112,481 bp, which is different from the QTL region in this study. Meanwhile, the copy number estimation using the ∆∆CT method also excluded the possibility of *TaDof* being the candidate gene for the QTL in our study (see below for details). These indicate that there are other candidate genes present in ‘Westonia’ for the PT phenotype.

The novel maker developed in this study was mapped to the adjacent region reported by Cook et al. (2004) (*gwm247, gwm340, gwm547,* and *BARC77*), Pan et al. (2017) (*gwm547–gwm247*) and Piñera‐Chavez et al. (2021) (gwm285 and *gwm547*). Furthermore, the physical region mapped with MutMap in the current overlapped with the region from those of Zhao (2019) (*NM1756*–*NM3950*). Six genes were selected based on BSR-seq analysis, differential expression analysis and gene functional annotation. The gene effect of *TraesCS3B02G597900* on PT phenotype was verified through an SNP maker in 171 historical wheat cultivars. These results demonstrate that *TraesCS3B02G597900* is the candidate gene underlying the 3BL QTL in our study.

### TraesCS3B02G597900 is a putative candidate gene for pith-thickness

Based on sequencing results, *TraesCS3B02G597900* (*TaVPE3cB*) showed a 9 bp Indel and multiple nonsynonymous SNPs between the two parents. RNA-seq results showed that this gene has significant differences between the two parents (log_2_FC = −3.77) and between the two extreme pools (log_2_FC = −2.55). In addition, qRT-PCR results confirmed that this gene was highly expressed in low PT samples during the stem elongation stage, which is a critical time for the central pith cells to initiate apoptosis and form the pith cavity (Nilsen et al. 2020). The negative correlation between gene expression level and pith thickness extent was confirmed in the current study. In addition, the expression profiles of *TaVPE3cB* in CS and 30 wheat varieties indicated that a 309 bp insertion in the promoter might inhibit the gene expression. This insertion contains a unique MBS motif that is related to drought inducibility. Several studies confirmed that gene expressions can be affected by large Indels located in the promoter region. For example, a 160-bp insertion in the promoter of *Rht-B1i-1* significantly enhances the gene expression and significantly increased the plant height of wheat (Lou et al. 2016). Recently, Mao et al. (2022) confirmed that a 108 bp insertion in the promoter of *TaNAC071-A* increases its gene transcription level and drought tolerance. However, further functional validation of this 309 bp indel in the promoter of *TaVPE3cB* is required.

We designed a dominant SNP marker *Qpt3B-F1/R1* and re-mapped it in the DH population and found the phenotype was co-segregated with it. When genotyping 171 historical Australian wheat cultivars, *TaVPE3cB.b* is significantly related to low pith thickness with a correlation coefficient of 0.501 (*P* < 0.01), and the ratio of *TaVPE3cB.a* to *TaVPE3cB.b* is about 2:1, suggesting that the percentage of high PT wheat variety is less than the low one. This finding is consistent with the fact that most wheat cultivars grown worldwide have a hollow stem with a thin pith, only a small number of varieties have fully developed stem pith cells and exhibit solid stemmed phenotype (Pluta et al. 2021).

Improving the pith thickness of wheat stem is a way to increase the ability of wheat to resist lodging (Kong et al. 2013), wheat stem sawfly (Beres et al. 2011), and drought stress (Monneveux et al. 2012). Several studies have shown that the diameter and the wall thickness of the basal stems are positively related to lodging and stem mechanical strength (Pinera-Chavez et al. 2016; Zuber et al. 1999). In addition, the thickness of the pith parenchyma also positively affects the mechanical resistance against stem bending. For instance, wheat cultivars with solidness-stems tend to have higher resistance against stem bending than hollow-stem wheat cultivars (Kong et al. 2013). However, stem wall thickness can lead to increasing stem material per unit of strength which can be biomass costly. Berry et al. (2007) suggested that the ideal strategy to enhance lodging resistance with the minimum biomass investment in winter wheat would be to increase internode width and internode material strength instead of stem wall thickness. Therefore, it might be a possible strategy of breeding lodging tolerance wheat with higher biomass through mutating *TaVPE3,* which might have some effects on the biosynthesis of the secondary cell wall and regulating pith thickness.

## Conclusion

The present study identified mRNA variants in common wheat for stem pith thickness through BSR-seq. One pith thickness-related candidate region was located on a 6.83 Mb physical interval of *Qpt-3B* using BSR-seq data. A total of sixteen genes were found differentially expressed, among them four DEGs, *TraesCS3B02G597800, TraesCS3B02G597900, TraesCS3B02G603900* and *TraesCS3B02G608500*, exhibited both differential expression levels and polymorphic SNPs between high PT and low PT samples. Finally, *TaVPE3cB* was identified as a high-confidence candidate gene for PT. The SNP makers for the candidate gene were developed and successfully separated *TaVPE3cB.a* and *TaVPE3cB.b* alleles. It was further applied to screen historical wheat cultivars of different pith thicknesses. In addition, an insertion in the promoter region of *TaVPE3cB* has been found related to the downregulation of this gene expression in wheat.

## Supplementary Information

Below is the link to the electronic supplementary material.

Supplementary file1 (PDF 1608 KB)

Supplementary file2 (XLSX 22262 KB)

## Data Availability

**Electronic supplementary material** The online version of this article (https://doi.org/xxxxx) contains supplementary material, which is available to authorized users.
