# Supplementary material for: The putative vacuolar processing enzyme gene TaVPE3cB is a candidate gene for wheat stem pith-thickness
Source: Theor Appl Genet. 2023 May 26;136(6):138. doi: 10.1007/s00122-023-04372-4 (PMC10219903; doi:10.1007/s00122-023-04372-4)
Supplement: Supplementary file 1 — Supplementary file1 (PDF 1608 KB) [file 122_2023_4372_MOESM1_ESM.pdf]

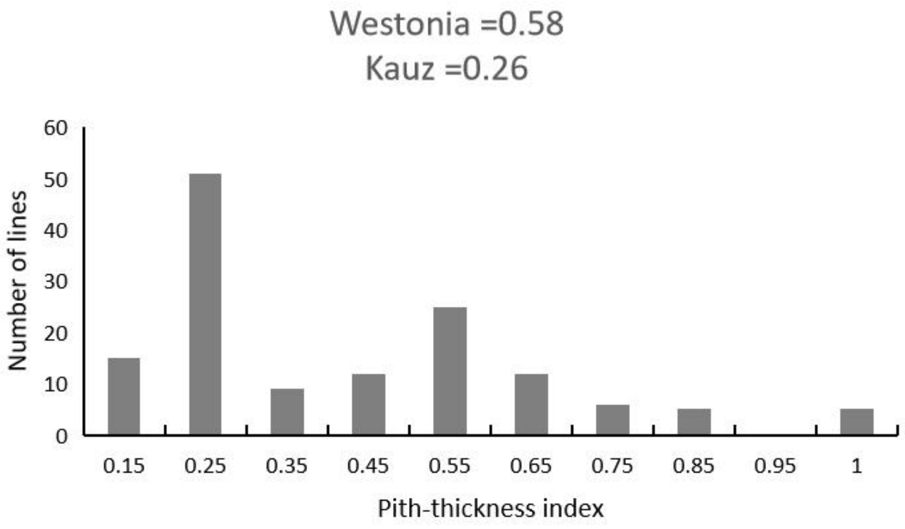

Fig. S1 Frequency distribution of PT index in the DH population. Scores are averaged across testing environments for DH population.

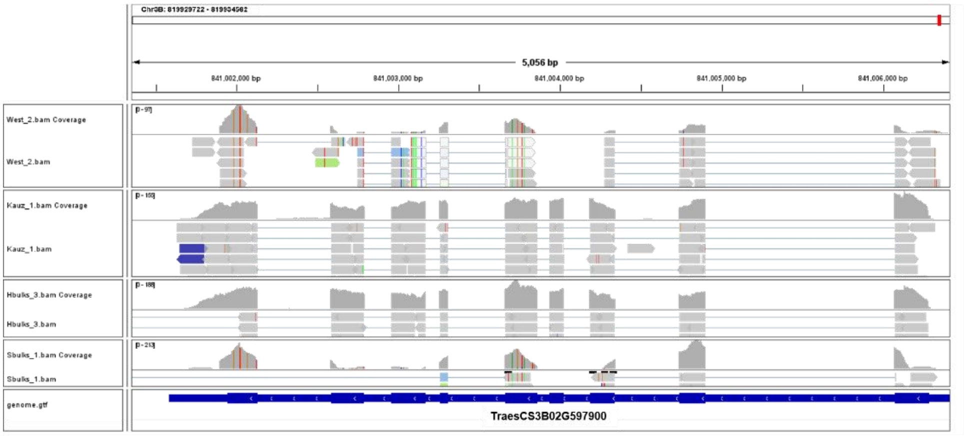

Fig.S2 Visualization of the mRNA sequence of candidate gene using IGV.

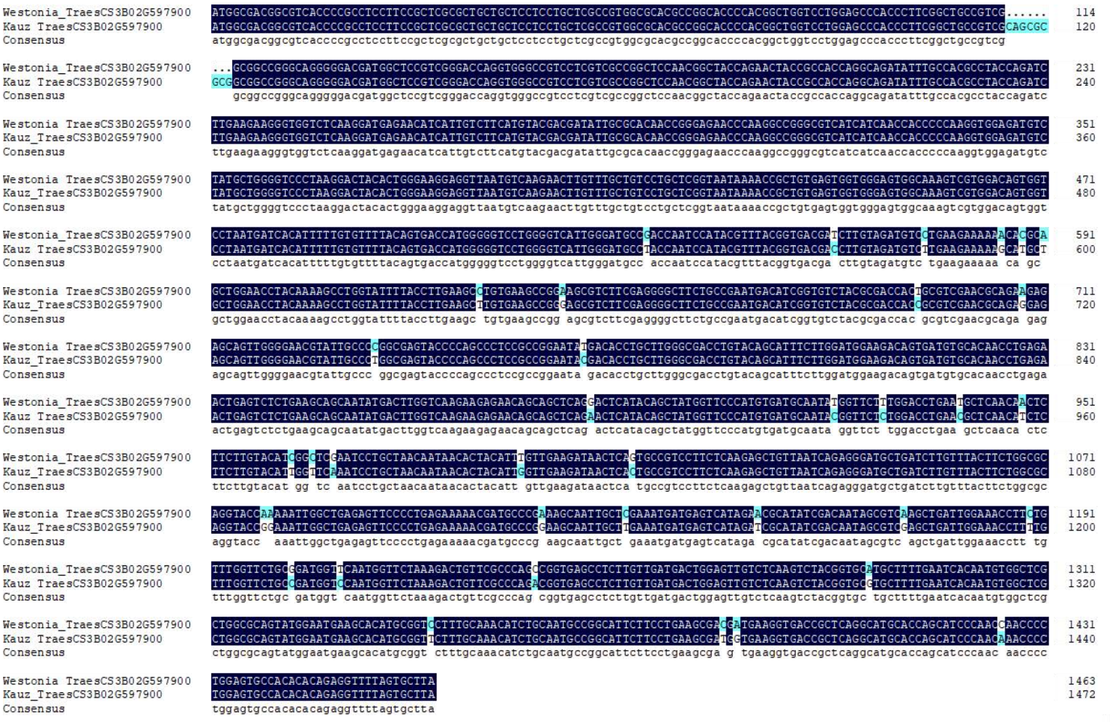

Fig.S3 Sequence alignment for CDS of *TraesCS3B02G597900* in 'Westonia' and 'Kauz'.

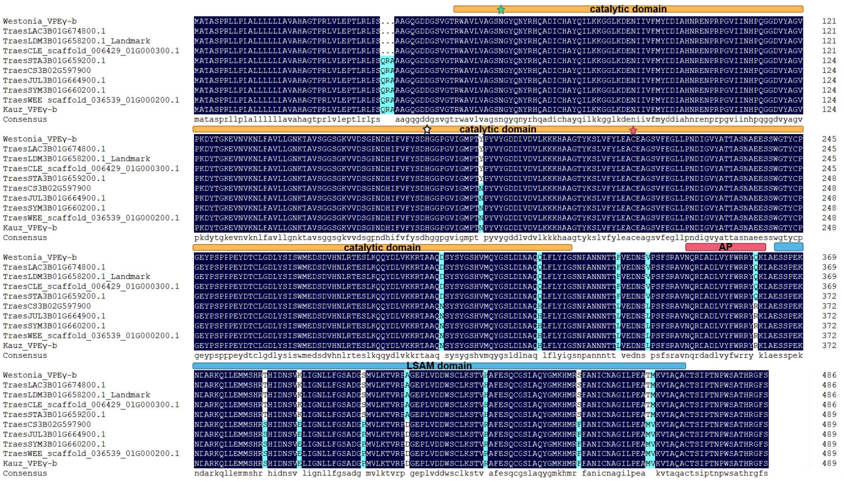

Fig.S4 Alignment of amino acid sequences of VPE gene (*TraesCS3B02G597900*) in ten common wheat varieties ('Westonia', 'Lancer', 'CDC Landmark', 'Claire', 'Janz', 'Chinese Spring', 'Julius', 'SyMattis', 'Weebill', 'Kauz'). The catalytic residues cysteine (red star), histidine (unfilled star), and asparagine (blue star) from the catalytic domain are shown.

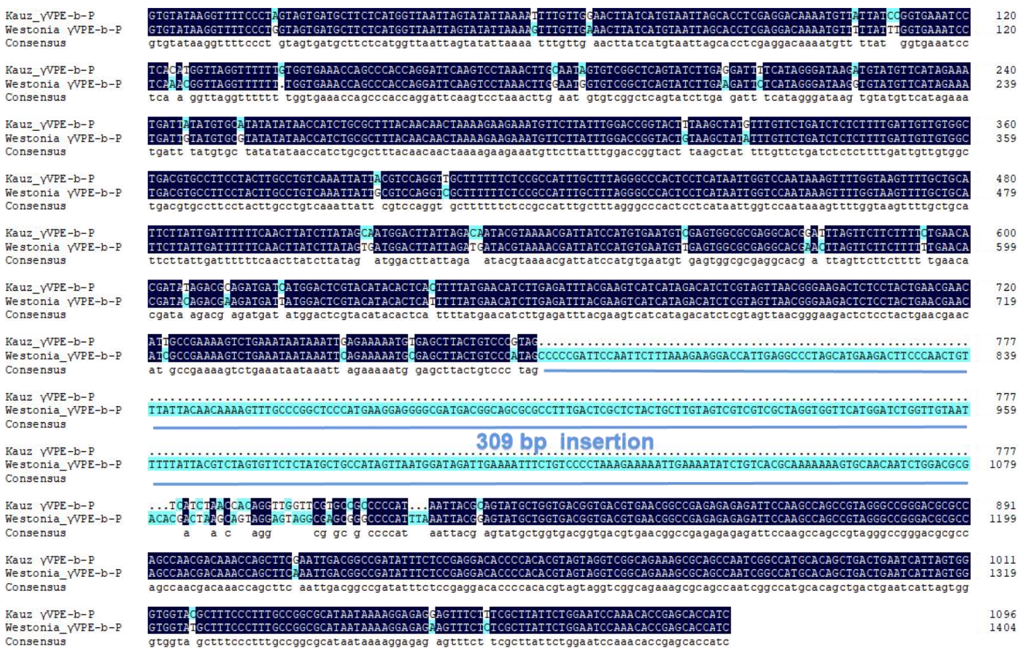

Fig.S5 Sequence alignment for promoters of *TraesCS3B02G597900* in Westonia and Kauz. Sequences on blue lines represent a 309 bp insertion.

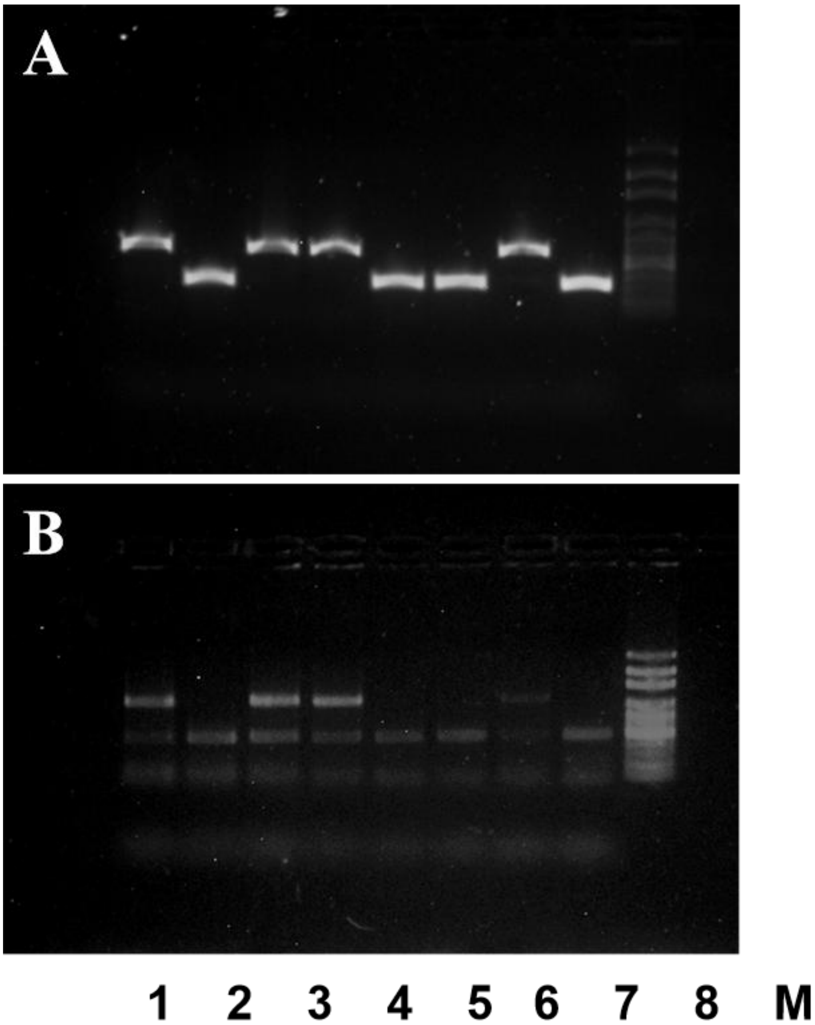

Fig.S6 Genotyping of Ta*VPE3cB* in different wheat cultivars.

(A) PCR with *Qpt3B-F2/R2*;

(B) PCR with *Qpt3B-F1/R1*. Lanes: M, molecular weight markers (Axygen® DNA LADDER 100BP), the bands are 3,000, 2,000, 1,000, 800, 700, 600, 500, 400, 300, 200 and 100bp, respectively; 1, 'Westonia' (*TaVPE3cB.b*), 2, 'Kauz' (*TaVPE3cB.a*), 3, 'Lancer' (*TaVPE3cB.b*), 4, 'Janz' (*TaVPE3cB.b*), 5, 'Chinese spring' (*TaVPE3cB.a*), 6, 'Stanley' (*TaVPE3cB.a*), 7, 'Landmark' (*TaVPE3cB.b*), 8, 'Filder' (*TaVPE3cB.a*).
